# Supplementary material for: Complexome profiling of the Chlamydomonas psb28 mutant reveals TEF5 as an early PSII assembly factor
Source: Plant Cell. 2025 Mar 17;37(6):koaf055. doi: 10.1093/plcell/koaf055 (PMC12164590; doi:10.1093/plcell/koaf055)
Supplement: koaf055_Supplementary_Data [file koaf055_supplementary_data.zip › psb28_interactive.zip!/plots/intensity/qualityPlots/peptides/PGRL1_Cre07.g340200.t1.1.html]

### Cre07.g340200.t1.1

Trivial name: PGRL1  
SeqCoverage [median]: 25.20  
Mass: 36.07 kDa  
  
 

| Sequence | MissedCleavage | Mass [Da] | wtIntensity | muIntensity | Score |
| --- | --- | --- | --- | --- | --- |
| ADMIFDEYK | 0 | 1130.50 | 0.00 | 792.63 | 108.98 |
| ADMIFDEYKR | 1 | 1286.60 | 37948.55 | 100046.38 | 136.96 |
| DNLILK | 0 | 714.43 | 0.00 | 6977.41 | 164.10 |
| FLEASMAYAK | 0 | 1129.55 | 95223.62 | 149054.62 | 189.70 |
| GKPIMTDEDYDALK | 1 | 1594.75 | 3108.06 | 7960.38 | 162.16 |
| GKPIMTDEDYDALKAELR | 2 | 2064.02 | 26815.39 | 282321.69 | 202.60 |
| KMYADAEPDYLR | 1 | 1470.68 | 23277.69 | 34255.86 | 82.54 |
| MYADAEPDYLR | 0 | 1342.59 | 39238.53 | 66148.40 | 209.60 |
| NKSSIVTAQGPR | 1 | 1256.68 | 12981.16 | 16438.05 | 126.66 |
| SKKMYADAEPDYLR | 2 | 1685.81 | 0.00 | 1258.55 | 45.28 |
| SSIVTAQGPR | 0 | 1014.55 | 139673.38 | 257438.69 | 262.84 |
| VVVVAETSEVK | 0 | 1158.65 | 17935.14 | 46521.51 | 179.81 |
| VVVVAETSEVKQEK | 1 | 1543.85 | 357925.31 | 484306.34 | 153.39 |
|  |  |  | 754126.8 | 1453520.5 |  |
